# Exploring the Necessity of Psychological Rounds and Psychological Interventions in General Hospitals in the Post-COVID-19 Period

**DOI:** 10.3389/fpsyg.2022.916776

**Published:** 2022-07-08

**Authors:** PeiXi Tang, MaoSheng Lee

**Affiliations:** ^1^Department of Sleeping Disorders and Neurosis, Brain Hospital of Hunan Province (The Second People’s Hospital of Hunan Province), College of Clinical Medicine, Hunan University of Chinese Medicine, Changsha, China; ^2^Department of Endocrinology, Shenzhen Traditional Chinese Medicine Hospital, The Fourth Clinical Medical College, Guangzhou University of Chinese Medicine, Shenzhen, China

**Keywords:** post-pandemic period, post-COVID-19 period, general hospital, psychological interventions, psychological rounds

## Abstract

**Objective:**

To explore the necessity of psychological rounds and psychological intervention in the post-COVID-19 period in a general hospital.

**Methods:**

Based on the current pandemic influence on Chinese people’s psychology, the medical experience, and environment were analyzed, and the feasibility of psychological evaluation and intervention were appraised with the psychological changes that might be brought by the medical behaviors, especially for surgical operations.

**Results:**

Nowadays, the pandemic is under full control in China, although the pandemic is rampant abroad. In China, the “Normalized pandemic prevention” phase has begun. In the post-COVID-19 period, the prolonged pandemic has made numerous people pessimistic, angry, and other negative emotions. Several general hospitals are facing huge influences: under the influence of anxiety, such as “higher hospital-acquired infection rate,” the patient attendance rate is reduced, and the hospital income is sharply reduced. Doctor–patient conflicts are more likely to occur during the medical procedures, affecting the medical experience, and reducing the rate of re-visit and referral.

**Conclusion:**

After analyzing a series of “endogenous” and “exogenous” factors of medical procedures in a general hospital in the post-pandemic period, it suggests that anxiety and depression caused by uncertainties in the medical procedures may be more obvious. Also, it is necessary to pay attention to the psychological status of patients and carry out psychological rounds and psychological interventions in general hospitals. The service quality can be improved, the medical experience can be ameliorated, and it can help general hospitals to turn “crisis” into “opportunity,” which also brings better development.

## Introduction

Since the end of January 2020, due to the spread of COVID-19, most Chinese citizens have suspended other major production activities other than basic living activities and stayed at home. Thanks to the joint efforts of the Chinese people, the pandemic in China has been effectively controlled. With the popularization of free vaccination for all, peoples’ life and work have returned to the status before the pandemic. As the pandemic abroad is still not under effective control, there have always been new imported cases appearing, and China’s pandemic prevention work has become normal. Therefore, in many peoples’ minds, medical institutions are still high-risk places and medical staff is more susceptible to infection, even under the effective pandemic prevention and control measures in China. They dare not go to the hospital for fear of infection by COVID-19 in a hospital or from contact with medical staff. They prefer to buy medicine in pharmacies in their way or get medicines online. For those who must be hospitalized, especially those who need surgery and other invasive operations, avoiding medical treatment is tantamount to giving up treatment. Therefore, they seem to be in a dilemma and will inevitably have more concerns and worries, resulting in anxiety and even depression. Based on the above special situation, this study attempts to explore the necessity of psychological ward rounds and psychological intervention in general hospitals in the post-pandemic period.

## Analysis of Psychological Factors Affecting Surgical Patients in General Hospitals

For many diseases, surgery has the effect of fundamentally and quickly solving the pain, but as an invasive operation, it has a certain trauma to the human body itself. Being diagnosed with a disease and being told that surgical treatment is needed can be regarded as a serious negative life event for patients. After diagnosis, most patients will have different degrees of anxiety, loneliness, inferiority complex, and other psychological events. Also, surgery and anesthesia are strong sources of stress, the curative effect of surgical trauma, uncertainty factors, the economic problems, and various aspects can all aggravate the patients’ negative psychology, thus, leading to the psychological stress. Patients may fall into various negative emotional reactions, including anxiety, tension, insomnia, fear, depression, etc., which may start from the preoperative, intraoperative, to postoperative period. These negative emotions affect patients’ vital signs, such as blood pressure and pulse, to varying degrees, and adversely affect the operation implementation and prognosis (Cao, 2011; Zheng et al., 2013). Taking anxiety as an example, it is an independent risk factor affecting the development and prognosis of the disease (Zhao et al., 2007). Anxiety can stimulate the thalamic-adrenal cortex system, excite the sympathetic nerve, increase the release of catecholamine, and excite the β receptor, resulting in accelerated heart rate, increased oxygen consumption of the myocardium, and increased blood pressure. Meanwhile, anxiety also stimulates the α receptor, causing coronary artery spasms (Chen et al., 2019), thus, significantly increasing the risk of surgery and anesthesia. At the same time, these negative emotions will reduce patients’ compliance and cooperation with surgery and postoperative rehabilitation.

Patients must bear not only the physical disease itself, including all kinds of physical discomforts, like a pain but also the psychosomatic distress caused by the negative emotions mentioned above. However, due to the great success of biological models in the control of acute diseases, doctors often ignore the above conditions by focusing only on biological factors in the diagnosis and treatment of diseases. The disease is born in the human body. As the “carrier” of the disease, people are often affected by various psychological and social factors. The emphasis on psychosocial factors is a new medical model of “bio-psycho-society” based on “mind-body monism,” which avoids the disadvantages of the old model of “seeing the disease and not seeing the person” and focuses on the “sick person” rather than the disease itself (Xu, 1999). Such a monistic model can help clinicians better understand patients and their diseases, not only promote disease recovery more comprehensively and shorten the average hospital stay, but also play a good role in building a harmonious doctor–patient relationship and greatly improve patient satisfaction.

Many medical institutions, both in China and abroad, have studied a lot of preoperative and postoperative psychological interventions (Powell et al., 2010; Davidson et al., 2016; Chew et al., 2020; Villa et al., 2020; Gorsky et al., 2021). Research from Tongji University, an affiliate of Shanghai Pulmonary Hospital, based on thoracoscope lung resection of 174 cases of patients with non-small cell lung cancer, found that the implementation of perioperative psychological support can improve the psychological statement of patients after they get into the operating room, which means it helps to reduce the stress, maintaining the vital signs, such as blood pressure and heart rate, etc., more stable. At the same time, the postoperative extubation time, the total length of hospital stay, and the incidence of postoperative complications were also significantly reduced (Yan and Wang, 2020), leading to the reduction of the psychological and economic burden of patients and improving patient satisfaction.

## Psychological Analyses of Non-Operative Patients in a General Hospital

**FIGURE 1:**
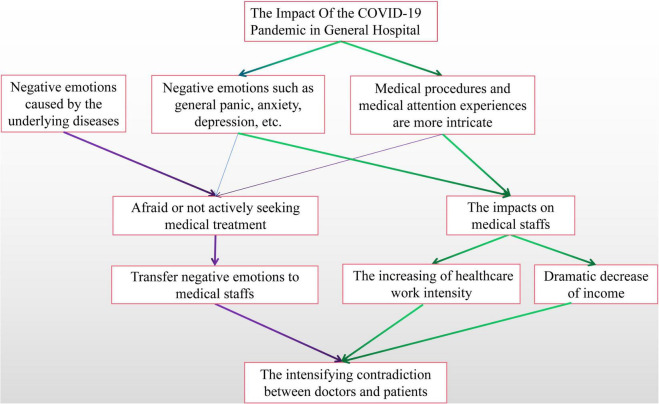
Analysis of psychological factors affecting patients in general hospitals.

Compared with non-surgical patients, patients requiring surgical treatment may suffer from greater psychological pressure. Psychological intervention during the perioperative period is conducive to the smooth implementation of surgery and postoperative rehabilitation of patients. Similarly, psychological ward rounds in non-surgical departments can also help to satisfy patients’ needs and grasp their psychological dynamics and promote mutual understanding between doctors and patients, and humanistic care for patients (Roditi and Robinson, 2011; Harvey, 2015; Tselebis et al., 2016). For example, diabetes, as a chronic disease, affects patients throughout their life. Diabetes is characterized by a long course of the disease, poor curative effect, and various complications. Not only physical pain, but it also causes serious mental damage. In recent years, a large number of domestic and foreign studies have shown that the prevalence of anxiety, depression, insomnia, and other mental and psychological disorders in patients with diabetes is significantly higher than that in the general population (Smith et al., 2013; Chew et al., 2015; van Son et al., 2015; Chaturvedi et al., 2019; Yu and Yang, 2019; Liu et al., 2020). As early as the early 1990s, some scholars proposed that psychological disorders associated with diabetes should be treated as a special serious complication (Gong et al., 1997). Currently, the diagnosis and treatment of diabetes-related complications still focus on the physical aspect, and the diagnosis and treatment of mental disorders associated are seriously inadequate. However, self-management at home is more difficult for diabetics than for other chronic diseases. Strict daily dietary intake, regular blood glucose monitoring, etc., makes every patient with diabetes under huge psychological pressure, completing the psychological intervention can make diabetics taking more actively implement self-management, and, moreover, achieve the strict control of blood sugar and avoid or delay the onset of other complications of both long-term and short-term treatment goals (Yin et al., 2021). Therefore, for a better description, the analysis of psychological factors affecting patients in general hospitals could be seen in the Figure 1.

## Psychological Analysis and Countermeasures of General Hospital Management and Patients’ Psychologic Status During the Post-Pandemic “Normalized Pandemic Prevention” Period

Since the outbreak of the COVID-19, the Chinese people have gone through the most severe periods, such as “home quarantine for all the citizens” and “medical staff who were, regardless of life-or-death situations, worked hard to help Hubei province, especially Wuhan City to fight the pandemic.” While the pandemic in China is under full control, it is getting more and more rampant abroad, the “normal pandemic prevention” phase has begun, during which we follow the policy that “preventing the importation from abroad and the rebound from home.” Then, come the current post-pandemic period of “regular nucleic acid testing for key populations” and “over 3.36 billion people were vaccinated (Bureau of disease control and Prevention, 2021).” Many hospitals are facing a huge impact, and the number of outpatients and inpatients decreased to a certain extent, compared with the same period in previous years (Huang and Xie, 2021; Lili and Guo, 2021). There may be at least two reasons for this: under the influence of anxiety, people worried about a higher probability of infection while being in the hospital, which made not only the number of visits has dropped, but the hospitals also have to set some full-time positions in daily pandemic prevention (Chew et al., 2020), resulting in a significant increase of hospital operating costs than usual, while the income was greatly reduced. Many small and medium-sized hospitals have been struggling to survive, the medical staff’s economic living standards have fallen, and due to the high risk of infection at work, medical staff also bear more pressure at work. The two reasons made their work enthusiasm somehow decline. On the other hand, the persistent and repeated pandemic makes both doctors (Wang, 2020) and patients have some negative emotions, such as pessimism, anger, and numbness. Doctor–patient conflicts are more likely to occur in the process of medical treatment, affecting patients’ medical experience, thus, reducing the rate of re-visit and referral, and making the survival of hospitals more difficult (Lili and Guo, 2021). These reactions are further highlighted by patients with a variety of negative emotional reactions before the pandemic.

**FIGURE 2:**
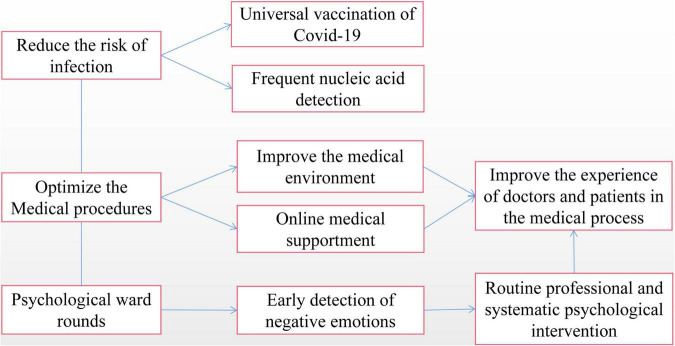
Psychological analysis and countermeasures of general hospital management and patients’ psychologic status during the post-pandemic “Normalized pandemic prevention” period.

To effectively alleviate the plight of both doctors and patients caused by the pandemic, major hospitals have taken many intervention measures. Some carry out hierarchical diagnosis and treatment, refine management, optimize hospitalization procedures, and transform a comfortable medical environment; some carry out online appointments and home continuous diagnosis and care through the internet, so that patients can get a certain degree of professional help at home and relieve the anxiety of possibly being infected with the virus by going-out activities. Some are offering more frequent nucleic acid tests to medical staffs, and encourage the staffs to get vaccinated against the COVID-19 to reassure the negative thoughts about high infection rates. Others provide psychological intervention and support to patients and their families or even medical staff. The measures mentioned above have alleviated the possible crisis of the hospital during the post-pandemic period to a certain extent and provided some comfort to the psychological of both doctors and patients. However, the above mentioned psychological interventions are usually provided only by general department nurses, who did not accept enough professional psychological knowledge, and they often have to shoulder the heavy general nursing work in the department at the same time, usually working on “three shifts,” which means they cannot supply sufficient professionalism, meaning regular and timely psychological service for eager patients who are struggling with nervousness needing stable supports. Routine psychological ward rounds, regular psychological intervention, and psychological crisis intervention under emergency conditions by qualified and systemically trained psychiatrists may be more worthy of consideration and praise. For better understanding, Psychological analysis and countermeasures of general hospital management and patients’ psychologic status during the post-pandemic “Normalized pandemic prevention” period could be seen in the Figure 2.

## Conclusion

In summary, during the post-pandemic period, it is necessary to pay attention to the mental and psychological status of patients. Launch psychological ward rounds and psychological intervention in general hospitals and reduce the negative aftereffects of the pandemic. Through psychological ward rounds and psychological intervention, patients’ medical concerns can be eliminated, service quality and medical experience for inpatients can be improved, doctor–patient conflicts can be reduced, and even general hospitals may be helped to turn “crisis” into “opportunity” in this special period, to better survive and even better develop. Perhaps, it will also be necessary to help hospitals out of this dilemma.

## Data Availability Statement

The original contributions presented in the study are included in the article/supplementary material, further inquiries can be directed to the corresponding author.

## Author Contributions

PT was mainly responsible for writing manuscript. ML was mainly responsible for the revision and guidance of the manuscript. Both authors agreed to be accountable for the content of the work.

## Conflict of Interest

The authors declare that the research was conducted in the absence of any commercial or financial relationships that could be construed as a potential conflict of interest.

## Publisher’s Note

All claims expressed in this article are solely those of the authors and do not necessarily represent those of their affiliated organizations, or those of the publisher, the editors and the reviewers. Any product that may be evaluated in this article, or claim that may be made by its manufacturer, is not guaranteed or endorsed by the publisher.
